# Multiple Beneficial Lipids Including Lecithin Detected in the Edible Invasive Mollusk *Crepidula fornicata* from the French Northeastern Atlantic Coast

**DOI:** 10.3390/md12126254

**Published:** 2014-12-22

**Authors:** Flore Dagorn, Florence Buzin, Aurélie Couzinet-Mossion, Priscilla Decottignies, Michèle Viau, Vony Rabesaotra, Gilles Barnathan, Gaëtane Wielgosz-Collin

**Affiliations:** 1 Faculté des Sciences Pharmaceutiques et Biologiques, Université de Nantes, Groupe Mer, Molécules, Santé—EA 2160, Institut Universitaire Mer et Littoral FR3473 CNRS, 9 rue Bias, BP 53508, F-44035 Nantes Cedex 1, France; E-Mails: flore.dagorn@gmail.com (F.D.); florence.buzin@orange.fr (F.B.); aurelie.couzinet-mossion@univ-nantes.fr (A.C.-M.); vony.rabesaotra@univ-nantes.fr (V.R.); Wielgosz-Collin@univ-nantes.fr (G.W.-C.); 2 Faculté des Sciences et des Techniques, Université de Nantes, Groupe Mer, Molécules, Santé—EA 2160, Institut Universitaire Mer et Littoral FR3473 CNRS, 2 rue de La Houssinière BP 92208, F-44322 Nantes Cedex 3, France; E-Mail: Priscilla.Decottignies@univ-nantes.fr; 3 UR1268, Biopolymères-Interactions-Assemblages, Institut National de la Recherche Agronomique INRA, F-44300 Nantes, France; E-Mail: Michelle.Viau@nantes.inra.fr

**Keywords:** the Bourgneuf Bay (Atlantic French coast), *Crepidula fornicata*, fatty acids, health benefits, invasive species, lecithin, mollusk, phospholipids, seasonal study, sterols

## Abstract

The invasive mollusk *Crepidula fornicata,* occurring in large amounts in bays along the French Northeastern Atlantic coasts, may have huge environmental effects in highly productive ecosystems where shellfish are exploited. The present study aims at determining the potential economic value of this marine species in terms of exploitable substances with high added value. Lipid content and phospholipid (PL) composition of this mollusk collected on the Bourgneuf Bay were studied through four seasons. Winter specimens contained the highest lipid levels (5.3% dry weight), including 69% of PLs. Phosphatidylcholine (PC) was the major PL class all year, accounting for 63.9% to 88.9% of total PLs. Consequently, the winter specimens were then investigated for PL fatty acids (FAs), and free sterols. Dimethylacetals (DMAs) were present (10.7% of PL FA + DMA mixture) revealing the occurrence of plasmalogens. More than forty FAs were identified, including 20:5*n*-3 (9.4%) and 22:6*n*-3 (7.3%) acids. Fourteen free sterols were present, including cholesterol at 31.3% of the sterol mixture and about 40% of phytosterols. These data on lipids of *C. fornicata* demonstrate their positive attributes for human nutrition and health. The PL mixture, rich in PC and polyunsaturated FAs, offers an interesting alternative source of high value-added marine lecithin.

## 1. Introduction

Coastal environments are essential habitats for many marine species of importance for human food and industry. Invasive non-native species are one of the crucial factors causing biodiversity loss and are often the cause of economic problems, mainly via interspecific interactions with commercially valuable species. They are thus a major concern in ecology and conservation of the coastal zones, mainly bays and estuaries [1,2,3,4]. The slipper limpet *Crepidula fornicata* is an example of an introduced species that may have huge effects on its environment. Native to the North American Atlantic coasts, this gastropod mollusk has been repeatedly introduced in Western Europe during the nineteenth and twentieth centuries, often due to imports and cultures of oysters [2]. These populations have reached spectacular densities in many European ecosystems, particularly in highly productive French ecosystems where shellfish are exploited [2,3,4,5]. For these regions, the potential negative effects of high densities of the suspension-feeder slipper limpet are especially acute [2,6,7]. Nowadays devoid of commercial value, *C. fornicata* mainly acts as a space and trophic competitor for valuable commercial species, such as the common scallop *Pecten maximus*, the mussel *Mytilus edulis* or the Japanese oyster *Crassostrea gigas* [2,7]. However, the diets of *Crepidula fornicata* and *Crassostrea gigas*, the overlap of their trophic niches and the existence of a competition between them have been shown to vary seasonally according to the availability of local food sources [7].

It is extremely difficult to control a marine organism once it becomes established. The attempts for a full-scale eradication of *C. fornicata* in the marine environment, by manual removal for example, seem very costly [8]. An attractive approach could be the exploitation of this harmful invasive species, which is available in large amounts along the coasts, if interesting substances were discovered in it.

Marine lipids, typically rich in *n*-3 polyunsaturated fatty acids (PUFAs), mainly eicosapentaenoic acid (EPA, 20:5*n*-3) and docosahexaenoic acid (DHA, 22:6*n*-3), have beneficial effects on human health [9,10,11]. Thus, these essential fatty acids (FAs) are known for their positive effects on reducing cardiovascular risk [12,13], on obesity and metabolic syndrome [13], and against cancer [14]. The impact of *n*-3 PUFAs on brain function and mental health have also been examined in recent reports: they are able to improve the mitochondrial function [15].

The demand for marine oils already exceeds the supply of traditional sources (fisheries), making important to find alternative sources of marine oils [9]. An interesting approach could be the exploitation of marine resources accessible and available in significant quantities as are coastal invasive species. The aim of this investigation was to determine the potential economic value of this invasive mollusk species. The present study was designed to clarify the content of lipid and phospholipid (PL) classes, and the FA composition, and to detect any compound with a potential interest in lipids of *C. fornicata*. Samples were collected at four seasons in order to determine the period at which the lipid and PL contents were optimal.

## 2. Results and Discussion

### 2.1. Lipid Content and Lipid Class Composition at the Four Seasons

Adults and young adults of the suspension-feeder mollusk *Crepidula fornicata* were collected from the same site, an oyster farming site in the Bourgneuf Bay (French Atlantic coast), through four seasons of the year. Total lipid contents (% of dried bodies without shells) and percentages of the main lipid classes of *C. fornicata* at the four seasons are reported in Table 1.

**Table 1:** Seasonal lipid content (% dry matter) and lipid class composition (% total lipids) of the mollusk *Crepidula fornicata*. Values are the mean of three replicates (mean ± s.d.); s.d., standard deviation.

Collection Season	Total Lipids	Non-Polar Lipids	Glycolipids	Phospholipids
Winter (January)	5.3 ± 0.3	21.9 ± 0.8	5.5 ± 0.1	69 ± 1
Spring (April)	2.7 ± 0.2	21 ± 2	14.7 ± 0.2	62 ± 2
Summer (July)	3.3 ± 0.3	30 ± 1	13 ± 3	56 ± 1
Autumn (November)	3.1 ± 0.1	28.0 ± 0.4	11 ± 2	61 ± 1

The lipid content was significantly higher in winter (January) than in the other seasons. Thus, total lipids accounted for 2.7% in spring (April) to 5.3% in winter, while the lipid content was almost identical in summer (July) and autumn (November) (3.3% and 3.1% respectively). Non polar lipids accounted for 21% (spring) to 30% (summer) of total lipids. No free FAs were detected. Free sterols were the only major component in the neutral lipids in all specimens. No marked seasonal variations were observed for glycolipid contents (around 12%) through the year excepted in winter with a value of 5.5%. The predominant lipid class in *C. fornicata* was PLs for the four seasons. The more important level of PLs was obtained for winter samples with 69% of lipids. Interestingly, these winter samples were also the richest ones for total lipids.

Lipid contents reported in studies of lipids and FAs in marine mollusks are generally low. The gymnosome *Clione limacina*, an abundant gastropod of the pelagic food web in polar waters, has been deeply studied for lipids and may contain up to a 56% of PLs of total lipids [16,17]. The total lipid content of the pearl oyster *Pinctada fucata martensii* was found very low in all culture conditions (0.4%–2.0%), similar to other *Crassostrea* oysters [18,19]. The total lipid content of the mussel *Perna viridis* ranged from 14.5/100 g of dried mussel powder in spring to 7.8/100 g in autumn [20]. This result is in agreement with the findings in *Mytilus edulis* [21]. Total lipids on a wet weight basis was 1.8/100 g in the New-Zealand green mussel *Perna canaliculus* and 1.2/100 g in the Tasmanian blue mussel *M. edulis* [22]. In fact, many studies were carried out on particular organs and tissues and not on entire bodies, thus limiting useful comparisons.

Most recently, characteristics of lipids and FAs of different parts of the gastropod *Turbo cornutus*, an important marine food resource in Japan, were published [23]. It is also well known that lipid levels and compositions are generally dependent of environmental conditions such as the season, the zone of harvest, and the phytoplankton resources available [24,25]. The proportion of the lipid classes varied according to the sampling period and this may be attributed to the differences of the natural diet. In addition, seasonal variations in lipid contents and FA compositions of adult bivalves are linked to the reproductive cycle.

### 2.2. Phospholipid Class Composition at the Four Seasons

Furthermore, the separation and quantification of PL classes, carried out by HLPC-ELSD, showed that the main PL class through the year was phosphatidylcholine (PC) at 63.9% to 88.9% of total PLs, the highest level being observed in autumn (Table 2).

**Table 2:** Seasonal phospholipid class composition of *Crepidula fornicata* (% phospholipids). Values are the mean of three replicates (mean ± s.d.); s.d., standard deviation. nd, not detected.

Phospholipid Class	Winter	Spring	Summer	Autumn
Cardiolipin	6.0 ± 0.1	8 ± 1	10.1 ± 0.3	6.0 ± 0.6
Phosphatidylethanolamine	11.7 ± 0.3	2.1 ± 0.1	2.9 ± 0.1	nd
Ceramide aminoethylphosphonate	15.8 ± 0.2	11 ± 2	9.6 ± 0.3	3.7 ± 0.4
Phosphatidylserine	1.0 ± 0.1	1.0 ± 0.1	nd	nd
Lysophosphatidylethanolamine	nd	nd	nd	1.5 ± 0.2
Phosphatidylcholine	63.9 ± 0.3	76 ± 1	70.0 ± 0.8	88.9 ± 0.1
Undetermined	1.6 ± 0.1	2.1 ± 0.7	7.3 ± 0.1	--

The greatest proportion of phosphatidylethanolamine was detected in winter (11.7% of total PLs) when it was not detected in autumn samples. Phosphatidylserine was found only in winter and spring samples at 1%. Phosphatidylinositol and diphosphatidylglycerol, the relatively usual minor components of marine invertebrates, were not found in the samples. Interestingly, significant levels of ceramide aminoethylphosphonate (CAEP) were observed in the PLs, up to 15.8% in winter. This phosphonosphingolipid is composed of an ethanolamine head group linked to a ceramide via a phosphono group (O–P–C) and is widely distributed among mollusks [26,27,28]. Hemocyte membrane lipids of the oyster *Crassostrea gigas* and the clam *Ruditapes philippinarum* contained high proportion of CAEP [29]. Thus, CAEP may have potential implications in hemocyte functions. High CAEP contents were also reported in the mussel *Crenomytilus grayanus* gills and muscles [30]. Interestingly, cardiolipin occurred in all seasons at significant levels (6%–10%). It is known as a unique mitochondrial PL, which was deeply studied in some marine bivalves [31,32]. Mammalian cardiolipin content and its PL FA composition in winter are of importance for optimal mitochondrial respiratory performance [32].

Taking into account that specimens collected in winter are the most interesting ones for lipid content and composition, these specimens were then investigated for PL FAs and free sterols as the major part of the neutral lipids.

### 2.3. Phospholipid Fatty Acid Composition in Winter

All FAs of the total PLs were converted into the fatty acid methyl esters (FAMEs) by transmethylation with methanolic hydrogen chloride. FAMEs are converted to *N*-acyl pyrrolidides in order to locate double bonds and branching. In addition to the FAMEs, the resulting mixture also contained nine fatty aldehyde dimethylacetals (DMAs). Mass spectra of the latter compounds showed characteristic intense fragment ion at *m*/*z* 75 ([(CH_3_O)_2_ − CH]^+^) and an ion corresponding to [M − 31]^+^ [27]. More than forty FAs with chain lengths between C_14_ and C_24_ were identified in PLs of winter samples. The PL FA and DMA composition is given in Table 3.

**Table 3:** Phospholipid fatty acids of *Crepidula fornicata* in winter samples. ^a^ ECLs (equivalent chain lengths) were determined using column CP-Sil 5 CB. *i*, *iso*; *ai*, *anteiso*; br, branched. Values are the mean of three replicates (mean ± s.d.); s.d., standard deviation.

Fatty Acids (Symbol)	ECL ^a^	Abundance (wt %)
Saturated Fatty Acids (SFAs)		
14:0	14.00	1.13 ± 0.02
4,8,12-Me_3_-13:0	14.49	1.18 ± 0.03
15:0	15.00	0.62 ± 0.04
*i*-16:0	15.60	1.04 ± 0.03
16:0	16.00	11.7 ± 0.6
*i*-17:0	16.64	2.20 ± 0.06
*ai*-17:0	16.73	1.36 ± 0.02
17:0	17.00	1.49 ± 0.08
*i*-18:0	17.64	0.34 ± 0.01
18:0	18.00	5.49 ± 0.09
br-20:0	18.38	0.24 ± 0.01
Total SFAs	--	27 ± 1
Monounsaturated fatty acids (MUFAs)		
9-16:1	15.74	1.90 ± 0.02
7-Me-8-16:1	16.12	0.21 ± 0.01
7-Me-6(*Z*)-16:1	16.20	0.22 ± 0.01
7-Me-6(*E*)-16:1	16.53	0.95 ± 0.05
9-18:1	17.72	4.2 ± 0.1
11-18:1	17.80	2.71 ± 0.09
11-20:1	19.68	4.85 ± 0.02
13-20:1	19.73	2.64 ± 0.05
br-21:1	20.32	0.64 ± 0.01
Total MUFAs		18.3 ± 0.3
Polyunsaturated fatty acids (PUFAs)		
18:4*n*-3	17.54	1.11 ± 0.04
18:2*n*-6	17.66	2.11 ± 0.01
20:4*n*-6	19.24	7.64 ± 0.03
20:5*n*-3	19.34	9.4 ± 0.1
20:2*n*-9	19.52	1.13 ± 0.04
20:2*n*-7	19.63	1.52 ± 0.02
22:6*n*-3	21.12	7.3 ± 0.3
22:4*n*-6	21.19	1.34 ± 0.03
22:5*n*-3	21.28	3.8 ± 0.1
22:3*n*-6	21.34	0.36 ± 0.02
22:2*n*-9,15	21.40	1.86 ± 0.01
22:2*n*-7,15	21.46	6.19 ± 0.08
Total PUFAs		43.8 ± 0.8
Fatty aldehyde dimethylacetals (DMAs)		
16:0	16.48	0.63 ± 0.02
br_1_-17:0	17.12	1.14 ± 0.02
br_2_-17:0	17.22	1.00 ± 0.04
17:0	17.48	0.47 ± 0.06
br_1_-18:0	18.10	0.95 ± 0.09
br_2_-18:0	18.22	0.24 ± 0.04
18:0	18.48	3.51 ± 0.05
br-19:0	19.22	0.24 ± 0.01
20:1	20.17	2.48 ± 0.03
Total DMAs		10.7 ± 0.4

Minor FAs as traces (<0.2%) (ECL): *i*-15:0 (14.62); *ai*-15:0 (14.69); 22:0 (22.00); 24:0 (24.00); 4-16:1 (15.70); 11-16:1 (15.82); *i*-9-17:1 (16.38); 9-17:1 (16.78); 2-OH-16:0 (17.18); *i*-19:0 (18.61); *ai*-19:0 (18.72); 21:1 (20.88); 5-24:1 (23.27); 22:2*n*-7, 13 (19.59).

The PL FA mixture was constituted by about 27% saturated FAs of the total FA + DMA mixture, 18.3% monounsaturated FAs, 43.8% of PUFAs, and 10.7% of DMAs. Saturated FAs included palmitic acid as the major one (11.7%) and a number of branched FAs, mainly *iso* and *anteiso* FAs. Monounsaturated FAs contained the 11-20:1 acid as major one and several mono branched FAs, including three branched 17:1 acids. Interestingly regarding their biological interest, PUFAs accounted for about 44%, the major ones being eicosapentaenoic 20:5*n*-3 (EPA, 9.4%), arachidonic 20:4*n*-6 (7.64%) and 22:6*n*-3 (DHA, 7.28%) acids. Due to their positive impact on human health, alternative sources of PUFAs are being pursued, including mainly marine sources [9]. Indeed, a number of studies indicate the importance of *n*-3 PUFAs, which offer various additional health benefits [12,13,14,15]. Also interesting from this point of view was the occurrence of the *n*-3 docosapentaenoic acid (DPA, 22:5*n*-3) (3.8%). Significant levels of DPA were also reported in organs of the gastropod *Turbo cornutus* [21]. A recent review showed beneficial effects of DPA, which is effective in the inhibition of platelet aggregation and has strong endothelial cell migration ability [33].

Three non-methylene-interrupted (NMI) diunsaturated C_22_ FAs were identified in PLs of *C. fornicata*, including 22:2*n*-7,15 acid at an unexpected high level (6.19%), and 22:2*n*-9,15 and 22:2*n*-7,13 at lesser amounts. Such C_22_ and C_20_ diunsaturated NMI FAs occur commonly in various mollusks and were also found in other marine invertebrates [28,34]. They are preferentially incorporated in polar lipids and their unusual unsaturation pattern confers to cell membranes a higher resistance to oxidative processes and microbial lipases than the common PUFA [34,35]. Another interesting result of this study was the identification of nine C_16_ and C_20_ fatty aldehyde dimethylacetals in lipids of *C. fornicata* at 0.2% to 3.5%. This finding points to the presence of plasmalogens (1-alkenyl-2-acyl ether glycerophospholipids). The biological role of plasmalogens in mollusks is not clearly known but it was proposed that they may have a role in the protection against oxidative stress [35,36,37,38]. Interestingly, plasmalogen forms of PLs were found to be specifically enriched with NMI FAs (22:2*n*-7,15 and 22:2*n*-9,15 acids and their precursors) in a recent investigation of lipids of three marine bivalves [35]. As for lipid and PL contents, seasonal variations of the FA composition of adult bivalves are closely linked to the reproductive cycle and affected by the availability and composition of the natural diet [24,25,26]. In addition, as for the FA determination of mollusk lipids, only some bivalve species, such as oysters and mussels, have been investigated in detail [16,17,18,19,20,21,22,23,27,28,29,30,31,33].

### 2.4. Free Sterol Composition in Winter

Free sterol fraction was the main part of neutral lipids (about 70%). Total free sterol composition of winter samples is given in Table 4.

**Table 4:** Composition of the free sterols of *Crepidula fornicata* in winter samples. X_1_, X_2_, X_3_, X_4_: unidentified sterols; Values are the mean of three replicates (mean ± s.d.); s.d., standard deviation.

Systematic Names	Trivial Names	% Sterol Fraction
24-*nor*-Cholesta-5,22*E*-dien-3β-ol	24-*nor*-Dehydrocholesterol	1.24 ± 0.07
24-*nor*-5α-Cholest-22*E*-en-3β-ol	24-*nor*-Dehydrocholestanol	0.69 ± 0.02
Cholesta-5,22*Z*-dien-3β-ol	22*Z*-Dehydrocholesterol	2.85 ± 0.04
Cholesta-5,22*E*-dien-3β-ol	22*E*-Dehydrocholesterol	8.33 ± 0.08
5α-Cholest-22*E*-en-3β-ol	22-Dehydrocholestanol	3.13 ± 0.03
5α-Cholest-5-en-3β-ol	Cholesterol	31.29 ± 0.04
5α-Cholestan-3β-ol	Cholestanol	7.21 ± 0.06
24-Methylcholesta-5,22*E*-dien-3β-ol	Brassicasterol/Crinosterol	18.6 ± 0.1
X_1_ (Δ° C_28:0_)	--	0.32 ± 0.01
24-Methylcholesta-5,24(28)-dien-3β-ol	24-Methylenecholesterol	7.32 ± .04
24-Methylcholest-5-en-3β-ol	Campesterol 22,23-Dihydrobrassicasterol	6.72 ± 0.08
X_2_ (Δ° C_29:2_)	--	0.54 ± 0.02
24-Ethylcholest-5,22*E*-dien-3β-ol	Poriferasterol/Stigmasterol	0.45 ± 0.02
24-Ethyl-5α-cholest-22*E*-en-3β-ol	Poriferastanol/Stigmastanol	0.43 ± 0.01
24-Ethylcholest-5-en-3β-ol	β-Sitosterol/Clionasterol	4.62 ± 0.07
24-Ethylcholesta-5,24(28)-dien-3β-ol	Fucosterol	5.13 ± 0.06
X_3_ (Δ° C_30:0_)	--	0.32 ± 0.03
X_4_ (Δ° C_30:0_)	--	0.37 ± 0.03
22,23-Methylene-23,24-dimethylcholest-5-en-3β-ol	Gorgosterol	0.38 ± 0.01

Cholesterol was the most abundant sterol at a relatively low level (31.29% of the total free sterol fraction). Other major sterols present were brassicasterol (18.6%), 22-dehydrocholesterol (8.33%), 24-methylenecholesterol (7.32%), cholestanol (7.21%), campesterol (6.72%), fucosterol (5.13%), sitosterol (4.62%). In a former study, it was shown that *C. fornicata* is able to synthesis sterols from acetate and the sterol composition was given [39]. Gorgosterol containing a side-chain cyclopropyl group was identified in the sterol mixture. It is found in some marine invertebrates, generally associated with symbiotic dinoflagellates [40].

Mollusks usually contain a number of different sterols with cholesterol present as the major sterol, mainly trans-22-dehydrocholesterol, brassicasterol, 24-methylenecholesterol, and campesterol [16,17,18,19,20,39]. Nine sterols were identified in *Perna viridis*, with cholesterol and desmosterol, brassicasterol and 24-methylenecholesterol as the main ones [20]. The intertidal limpets *Cellana grata* and *Cellana toreuma* were shown to contain eleven different sterols, including cholesterol as the predominant component, and Δ8-sterols, namely zymostenol and zymosterol, present in only the male gonads at unusually high levels [41].

The 24-methyl- and 24-ethylsterols, known as phytosterols, such as campesterol, brassicasterol, sitosterol and stigmasterol, have well-established lowering-cholesterol effects [42,43,44,45]. They decrease the intestinal absorption of cholesterol. All phytosterols in the human body originate from the diet because humans cannot synthesize phytosterols. Furthermore, the action of phytosterols on cardiovascular diseases and the potent anti-inflammatory properties have been reported [45]. Thus, sterols from *C. crepidula* are of interest in that contain more than 40% of phytosterols.

## 3. Experimental Section

### 3.1. Specimen Collection

*Crepidula fornicata* (L.) specimens (mean linear shell length ± s.d. = 4.2 ± 0.9 cm) were hand-collected in January, April, July and November 2007 at the oyster farming site of La Couplasse in the Bourgneuf Bay (French Atlantic coast, 46–47° N, 1–2° W) and immediately transported to the laboratory where they were cleaned of epibionts. For each season, the soft tissues were separated from the shells, pooled, homogenized with a blender, and then divided into three lots. Samples were stored at −20 °C before next treatment step.

### 3.2. Chemicals

Standards of PLs (cardiolipin, phosphatidylcholine PC, phosphatidylethanolamine, phosphatidylglycerol, phosphatidylinositol, phosphatidylserine, lysophosphatidylcholine, lysophosphatidylethanolamine, sphingomyelin) were purchased from Sigma-Aldrich (Saint-Quentin Fallavier, France). An authentic ceramide aminoethylphosphonate (CAEP) was kindly donated by Yanic Marty (CNRS, UBO, Brest, France).

### 3.3. Lipid Extraction and Separation of Lipid Classes

For each sample, homogenized fleshes (about 300 g fresh) were steeped in dichloromethane/methanol (1:1, v/v) for 3 h at room temperature. The extract was filtered into a Büchner funnel and washed with distilled water. After adding 0.01% w/w of BHT (butylated hydroxytoluene) as antioxidant, all extracts were stored at −20 °C under nitrogen before analysis. These organic extracts were then evaporated to provide crude total lipids, which were chromatographed on an open silica gel column (290 mm × 25 mm, 60 Å, 35–75 μm) with dichloromethane (neutral lipids), acetone (glycolipids) and methanol (PLs) as successive eluents as previously described [46].

### 3.4. Phospholipid Determination by HPLC-ELSD

High-performance liquid chromatography (HPLC) analysis of PLs was performed as previously described [46]. Briefly, a modular UltiMate. 3000 RS HPLC System (Dionex, Villebon sur Yvette, France) coupled to an evaporative light scattering detector (ELSD) Sedex 85 (Sedere S.A., Alfortville, France) was used. The ELSD was heated to 50 °C and nebulizer gas pressure (dried and filtered air) was maintained at 3.5 bar. The separation was achieved using a silica column Prevail™ (150 mm × 4.6 mm, 3 μm particle diameter—Alltech. Associates Inc., Lokeren, Belgium) heated at 25 °C with the flow rate set to 1.5 mL/min. Elution was conducted with (A) chloroform and (B) methanol/28% ammonia in water/chloroform (92:7:1, v/v/v). The gradient started at 0% B, increased to 20% in 3 min, then increased to 100% in 9 min and held for 3 min. Samples were dissolved in chloroform (2 mg/mL) and maintained at 10 °C. The injection volume was 10 μL. The lipids extracted were injected three times. Unknown concentrations of PLs were determined using standards curves calculated from values obtained by injecting 10 μL of chloroform serial diluted solutions of cardiolipin, PE, PC, SPH, LPC and LPE (1–10 μg), and CAEP, PI and PS (0.5–5 μg). The sum of PL concentrations was regarded as total PL concentration. Phospholipid species were expressed as a percentage of total PLs.

### 3.5. Preparation of Fatty Acid Methyl Esters, N-Acyl Pyrrolidides and Sterol Acetates

Fatty acid methyl esters (FAMEs) were obtained by PL transmethylation (40 min at 80 °C under reflux with 6% methanolic hydrogen chloride). *N*-Acyl pyrrolidide derivatives (NAPs) were prepared by direct treatment of the FAMEs with pyrrolidine/acetic acid (5:1, v/v) for 60 min at 85 °C under reflux [46]. Free sterols were isolated during lipid class separation from fractions eluted with dichloromethane and were converted into sterol acetates (SAs) by reaction with acetic anhydride and dried pyridine, during 12 h in darkness, at room temperature [47].

### 3.6. Gas Chromatography-Mass Spectrometry Analyses

FAMEs, NAPs and SAs were analyzed using an Agilent model 689 series II gas chromatograph linked to an Agilent 5973 series network mass selective detector (E.I. at 70 eV) equipped with an Agilent model 5973N selective quadrupole mass detector (Agilent, Les Ulis, France). Separation was achieved with a CP-Sil 5 CB low bleed MS capillary column (60 m × 0.25 mm I.D., 0.25 μm phase thickness—Chrompack, Middelburg, The Netherlands). Helium was used as the carrier gas under a constant flow rate (1 mL/min). For FAME analyses, the oven temperature was programmed at 170 °C during 4 min then allowed to drop to 300 °C at a rate of 3 °C/min. For NAP analyses, the oven temperature was programmed at 200 °C during 4 min then allowed to 310 °C at a rate of 3 °C/min, and maintained at this temperature for 20 min. For SA analyses, the oven temperature was programmed from 200 to 310 °C at a rate of 3 °C/min and then maintained for 25 min.

### 3.7. Statistical Expression of Data

All data presented are means more or less standard deviation (*n* = 3).

## 4. Conclusions

Finally, *C. fornicata* constitutes an alternative promising source of marine lecithin and other value-added lipids with nutritional and biomedical interest. Originally used to describe specifically PC mixtures, the term lecithin is, nowadays, commonly used to refer to commercial mixtures of different PLs. Since PLs are amphipathic molecules, lecithin of various compositions is commonly used in the food industry as an emulsifier. Thus, commercially available lecithin does not contain only PLs and their lysophosphatidyl derivatives, but also various glycolipid species and even neutral lipids [48,49]. Lecithin is usually isolated on an industrial scale from plant sources. However, soybean is currently the predominant commercial source of lecithin. Nevertheless, the latter lecithin is devoid of long chain *n*-3 PUFAs.

The biological and nutritional function and interest of lecithin have been the focus over recent researches, especially if rich in long-chain PUFAs. In this case, nutritional and pharmaceutical applications could be found [50,51]. It was recently showed that marine PC rich in *n*-3 PUFAs suppresses 1,2-dimethylhydrazine-induced colon carcinogenesis in rats by inducing apoptosis, thus suggesting an effective dietary protective factor against colon cancer [52]. Beneficial effects of marine lecithin dietary have also been mentioned in the treatment of psoriasis [53].

This study is the first report showing the potential of *C. fornicata* as an alternative source of marine lecithin rich in PC and long-chain *n*-3 PUFAs, especially for samples collected in winter (2.3 g of PC/100 g dry weight of fleshes) (Table 5).

**Table 5:** Candidates as a functional lipid from *Crepidula fornicate*.

Lipids	Biological Effects	References *
Lecithin (polyunsaturated FAs)	Protective factor against colon cancer	[50,51,52]
	Treatment of psoriasis	[53]
CAEP	Implications in some hemocyte functions	[31]
Cardiolipin	Optimization of mitochondrial respiratory performance	[34]
*n*-3 Polyunsaturated FAs (EPA, DHA)	Human health benefits	[9,10,11,12,13,14,15]
*n*-3 Docosapentaenoic acid (DPA)	Inhibition of platelet aggregation	[35]
	Influence to endothelial cell migration ability	[35]
Diunsaturated NMI FAs	Resistance to oxydative stress and microbial lipases	[36,37]
Plasmalogens	Anti oxydative stress	[37,38,39,40]
Phytosterols	Lowering cholesterol effects	[44,45,46,47]
	Action on cardiovascular disease	[47]
	Anti-inflammatory properties	[47]

* Citation from the text. CAEP: ceramide aminoethylphosphonate; NMI: non-methylene-interrupted.

In addition, other compounds identified in this mollusk are also candidates as functional lipids (Table 5). Moreover, *C. fornicata* is not only low in cholesterol but also contains a relatively high level of cholesterol-lowering phytosterols and of *n*-3 PUFAs (Table 5). It shows that this invasive mollusk, available at high abundance levels in several bays of the French Atlantic coast, may constitute an alternative source of value-added molecules with nutritional and biomedical interest such as lecithin and *n*-3 PUFAs.

## Abbreviations

CAEP: ceramide aminoethylphosphonate
DHA: *n*-3 docosahexaenoic acid
DMA(s): dimethylacetal(s)
DPA: *n*-3 docosapentaenoic acid
ELSD: evaporative light scattering detector
EPA: *n*-3 eicosapentaenoic acid
FA(s): fatty acid(s)
FAME(s): fatty acid methyl ester(s)
GC-MS: gas chromatography-mass spectrometry
NAP: *N*-acyl pyrrolidide(s)
NMI: non-methylene-interrupted
PC: phosphatidylcholine
PUFA(s): polyunsaturated fatty acid(s)
PL(s): phospholipid(s)
SA(s): sterol acetate(s)
